# Hydrodynamics-based liver transfection achieves gene silencing of CB1 using short hairpin RNA plasmid in cirrhotic rats

**DOI:** 10.1371/journal.pone.0228729

**Published:** 2020-02-13

**Authors:** Adriana Díaz-Rivera, Alejandra Meza-Ríos, Victoria Chagoya de Sánchez, Gabriela Velasco-Loyden, Leonel García-Benavides, Luis F. Jave-Suarez, Hugo Christian Monroy-Ramirez, Arturo Santos-García, Juan Armendáriz-Borunda, Ana Sandoval-Rodríguez

**Affiliations:** 1 Institute of Molecular Biology in Medicine, Centro Universitario de Ciencias de la Salud, Universidad de Guadalajara, Guadalajara, Mexico; 2 Tecnologico de Monterrey, Campus Guadalajara, Guadalajara, Mexico; 3 Cellular Physiology Institute, Universidad Nacional Autónoma de México, Mexico City, Mexico; 4 Biomedical Sciences Department, Centro Universitario de Tonala, Universidad de Guadalajara, Tonala, Mexico; 5 Immunology Division, Centro de Investigación Biomédica de Occidente, Instituto Mexicano del Seguro Social; National Institutes of Health, UNITED STATES

## Abstract

**Background:**

There is a correlation between the endocannabinoid system and hepatic fibrosis based on the activation of CB1 and CB2 receptors; where CB1 has profibrogenic effects. Gene therapy with a plasmid carrying a shRNA for *CB1* delivered by hydrodynamic injection has the advantage of hepatic tropism, avoiding possible undesirable effects of CB1 pharmacological inhibition.

**Objective:**

To evaluate hydrodynamics-based liver transfection in an experimental model of liver cirrhosis of a plasmid with the sequence of a shRNA for CB1 and its antifibrogenic effects

**Methods:**

Three shRNA (21pb) were designed for blocking CB1 mRNA at positions 877, 1232 and 1501 (pshCB1-A, B, C). Sequences were cloned in the pENTR^™^/U6. Safety was evaluated monitoring CB1 expression in brain tissue. The silencing effect was determined in rat HSC primary culture and CCl_4_ cirrhosis model. Hydrodynamic injection in cirrhotic liver was through iliac vein and with a dose of 3mg/kg plasmid. Serum levels of liver enzymes, mRNA levels of TGF-β1, Col IA1 and α-SMA and the percentage of fibrotic tissue were analyzed.

**Results:**

Hydrodynamic injection allows efficient *CB1* silencing in cirrhotic livers and pshCB1-B (position 1232) demonstrated the main CB1-silencing. Using this plasmid, mRNA level of fibrogenic molecules and fibrotic tissue considerably decrease in cirrhotic animals. Brain expression of CB1 remained unaltered.

**Conclusion:**

Hydrodynamics allows a hepatotropic and secure transfection in cirrhotic animals. The sequence of the shCB1-B carried in a plasmid or any other vector has the potential to be used as therapeutic strategy for liver fibrosis.

## Introduction

Hydrodynamics-based transfection [1, 2] presented new opportunities in RNAi field [3]. This procedure has been widely used for transfecting molecules to liver due to the marked gene expression achieved [4–6]. However, not many studies reported hydrodynamics use for cirrhotic livers. Also, hydrodynamics had been performed mostly in rodent tail-vein, but some authors have used large veins maintaining the same efficiency. Regarding silencing molecules, hydrodynamics had demonstrated proved effectiveness in PDGFR-β and HBx [7, 8]. Endocannabinoid system is constituted by endogenous cannabinoids and CB1 and CB2 receptors [9]. This system plays an important role in hepatic fibrogenesis and several studies have showed that CB1 receptor is overexpress in hepatic fibrosis, liver cirrhosis, NASH [10] and NAFLD [11, 12]. CB1 agonism has demonstrated a profibrogenic effect while its pharmacological or genetic inhibition reduces liver fibrosis [13, 14]. However, the drug Rimonobant had been banned for FDA since its use to treat obesity caused behavioral effects associated to depression and suicide intents [15]. Using iRNA mechanisms, *CB1* (also known as *CNR1*) gene silencing can reduce liver fibrosis in knockout models and without the undesirable side effects of pharmacological antagonism. Gene silencing can be achieved from expression vectors that code for short hairpin RNA (shRNA) capable of target specific mRNAs [16, 17]. Hepatic fibrosis is the response to chronic injury characterized for hepatic stellate cells activation that increase expression of fibrogenic molecules such TGF-β1 and Col I leading to histological changes in liver tissue due to accumulation of extracellular matrix proteins (EMC) [18]. To date there is not an established therapy for hepatic fibrosis, then we believe *CB1* silencing using shRNA launched by hydrodynamics transfection could exert important antifibrogenic effects, time-limiting the transfection to liver and avoiding its brain transfection. To evaluate this, three shRNA sequences to align rat *CB1* mRNA were designed and gene and protein silencing was tested in a cirrhosis model. We used a modification of the hydrodynamic transfection method [2, 19, 20] using iliac vein as administration via.

## Materials and methods

### Design and synthesis of shRNAs for CB1

Three sequences 21pb long for shRNAs targeting rat *CB1* mRNA (Accession #: NM_012784) were designed using Block iT RNAi software (Invitrogen). These shRNAs have a specific 5’-end to facilitate directional cloning into the pENTR ^™^/U6 plasmid as described in Table 1. All designed shRNA sequences were searched in Blast database rat genome to avoid off-target effect. Control shRNA irrelevant for rat genome was taken from a publication of Jin-Wook Kim [21]

**Table 1:** Sequences of the shRNAs designed for CB1 mRNA.

shRNACB1-A	Sense 5´**CACC**GGACTATCGCAATAGTAATCG**CGAA**CGATTACTATTGCGATAGTCC 3´ Antisense 5´**AAAA**GGACTATCGCAATAGTAATCG**TTCG**CGATTACTATTGCGATAGTCC 3´
shRNACB1-B	Sense 5´**CACC**GCTTGCGATCATGGTGTATGA**CGAA**TCATACACCATGATCGCAAGC 3´ Antisense 5´**AAAA**GCTTGCGATCATGGTGTATGA**TTCG**TCATACACCATGATCGCAAGC 3´
shRNACB1-C	Sense 5´**CACC**GCATCAAGAGCACCGTTAAGA**CGAA**TCTTAACGGTGCTCTTGATGC 3´ Antisense 5´**AAAA**GCATCAAGAGCACCGTTAAGA**TTCG**TCTTAACGGTGCTCTTGATGC 3´
shRNA-Irre	Sense 5´**CACC**GAATTCTCCGAACGTGTCACGT**CGAA**ACGTGACACGTTCGGAGAA 3´ Antisense 5´**AAAA**TTCTCCGAACGTGTCACGT**TTCG**ACGTGACACGTTCGGAGAATTC 3´

**Bold letters** indicate complementary sequences to pENTR/U6 and loop sequence

### Cloning of shRNA in expression vector

shRNA-CB1 sequences were cloned in pENTR^™^/U6 (Invitrogen) which has U6 promoter to be transcribed by RNA polymerase III. Generation of shRNA-CB1 sequences involved the alignment of two synthetic complementary oligonucleotides to generate a double-strand shRNA. T4 DNA ligase (Invitrogen, CA, USA) was used for 2 hours at 25°C for cloning the shRNA-CB1A, B, C and Irre into pENTR^™^/U6; followed by amplification in TOP10 competent cells (Invitrogen, CA, USA) and purification of the plasmids using QIAGEN Plasmid Mini Kit (QIAGEN). In order to verify correct cloning, sequencing of plasmids was performed using a U6 forward-primer: 5-GGACTATCATATGCTTACCG-3´.

### Rat HSC primary cultures

Freshly isolated Hepatic stellate cells (HSC) from rats were obtained according to the method of digestion with collagenase/pronase. Briefly, the livers of Wistar rats were perfused with 80 mg/20 mL of pronase for 17 min at 37°C and then with 6mg/20 mL of collagenase for 17 min at 37°C, both dissolved in GBSS Buffer with calcium. Subsequently, the liver was macerated in a DNase solution (10mg/mL) at 37°C, the resulting suspension was filtered through sterile gauze and centrifuged twice at 700 rpm/1min/15°C. The recovered supernatant was centrifuged 10 minutes/2700 rpm/15°C. In this step hepatic stellate cells were collected and ultracentrifuged in an Accudenz (Accurate Chemical) density gradient. Isolated HSC cells were recovered from the interphase and cultivated in DMEM containing 10% FBS (Multicell Wisent Inc).

### HSC transfection of shRNAs

Freshly isolated HSC were seeded in 10 cm dishes at a density of 3.6–4.9 x10^5^cells/well. After 7 days post-isolation 200,000 cells per well were seeded in 6 wells dishes. Cultures were transfected using 1.5 ul of Fugene® HD and 250 ng of the correspondent pshRNA. Control group had any plasmid. Cells were harvested at 48h. Confocal imaging was performed in a ZEISS LSM 800 with Airyscan confocal laser scanning microscope at Plan-APO 40x/ 1.3 Oil DIC III objective. The maximum projection and intensity of the fluorescence was analyzed with ZEN 2.3 SP1 software.

### Real time qPCR

RNA was isolated from cells or tissue using Trizol® Reagent (Invitrogen). Retrotranscription was made using 2μg of total RNA using M-MLV reverse transcriptase (Invitrogen) according to manufacturer´s instructions. qPCR using TaqMan probes for *CB1*, *TGFβ-1*, *Col IA1*, *α-SMA* (also known as *ACTA2*) and *GAPDH* with Universal PCR Master Mix (LifeTechnologies) was performed in a Rotor-gene equipment (Corbett research) following standard conditions: 2´hold 50°C, 5´hold 94°C, and 40 cycles of 30 sec at 94°C and 40 sec at 60°C. Sequences and catalog number of probes used are depicted in Table 2. Data were analyzed using 2^-ΔΔCt^ method.

**Table 2:** Probes for RT-qPCR.

Catalog number	Gene	Probe Sequence
Rn00572012_m1	TGFβ-1	ACCGCAACAACGCAATCTATGACAA
Rn01759928_g1	ACTA2	ACGTACAACTGGTATTGTGCTGGAC
Rn00670303_g1	Col IA1	GAGCTGCTGGCCCATCTGGCCTAA
Rn00562880_m1	CNR1	CCATGGCTGAGGGTTTCCCTCCCGGA
Rn99999916_s1	GAPDH	AAACCCATCACCATCTTCCAGGAGC

### Western blot

50 μg of total protein were separated by electrophoresis in a SDS-PAGE gel under denaturing conditions. Proteins were transfer to PVDF membranes (Bio-Rad Laboratories). Non-specific binding was blocked using a pre-incubation step of the membrane in PBS-Tween 0.1% + 1% BSA for 1 hour. Blotting for CB1 was at 1:1000 dilution (Thermo) while GAPDH was used as loading control (1:1000). Primary antibodies were detected using secondary antibodies that are biotin-labeled (1:5000; BM Chemiluminescence Western Blotting Kit Mouse/Rabbit, Roche Diagnostics). Image capture was achieved with ChemiDoc XRS+ imaging system (Bio-Rad Laboratories) and analysis of blots was performed using Image-lab 3.0 software (Bio-Rad Laboratories).

### Animal model of hepatic fibrosis

48 male Wistar Kyoto rats were randomly distributed into healthy control group and cirrhotic groups (n = 8). All the animals at the onset of CCl_4_ intoxication weighed about 120 g and received an intraperitoneal injection of a mixture of CCl_4_: mineral oil, three times per week for a period of 8 weeks. Male Wistar rats had free access to food and water (*ad libitum*). The animals were housed in polypropylene cages (four per cage) in a room under controlled temperature (22 ± 1°C) with 12 h light-dark cycles. The health of the animals was monitored 3 times per week. When animals were identified in pain during the CCl_4_ intoxication, had ear infection, showed slow or no-movement, had brittle hair and eye dehydration; animals were euthanized. Animal were obtained from the Animal facility of the Health Sciences University Center of the University of Guadalajara. Rats received care according to the Mexican Official Norm NOM-060-ZOO-1999 and guidelines of the Animal facility of the Health Sciences University Center of the University of Guadalajara. The protocol was approved by the Research and Ethical Committees of University of Guadalajara (approval number C.I. 67–2012) which reviewed and approved the animal care and sacrifice methods.

### Hydrodynamics administration of shRNA-CB1

Cirrhotic animals were transfected with 3 mg/kg of pshCB1-A, B, C, Irre or vehicle (n = 8, per group). The hydrodynamic injection was carried out in the iliac vein, with a total volume of 4 ml of plasmid solution for 5–7 seconds, animals were anesthetized with 80 microliters ZOLETIL 50 via i.p. Rats were sacrificed 96 hours post-administration of the shRNA-CB1 plasmid using an excess of anesthesia (150 microliters of ZOLETIL 50 via ip). Blood samples were taken for determination of serum liver enzymes (AST, ALT and albumin). Representative fragments of the five liver lobes were collected and immediately frozen at -80°C until molecular. For histological analysis, fragments of the three major liver lobes were fixed in 4% paraformaldehyde and embedded in paraffin. Brain was collected to monitor *CB1* expression.

### Liver morphometric analysis and liver enzyme determination

Paraffin embedded tissue was cut into 4 mm thick and stained with Masson's Trichrome. The percentage of fibrosis was quantified using Image ProPlus software in twenty photographs of microscopic fields. Fibrosis percentage was calculated as percentage of blue-stained tissue (MEC) in relation to the total tissue. AST, ALT and albumin serum levels were measured in an ERBA automatized analyzer.

### Statistical analysis

Quantitative data are expressed as the mean ± standard deviation. For comparison of non-parametric variables Kruskal-Wallis and Mann Whitney U test were used. p<0.05 was taken as significant. For statistical analysis the software GraphPad prism version 4.0 was used.

## Results

### Phenotype of hepatic stellate cells

**Fig 1:**
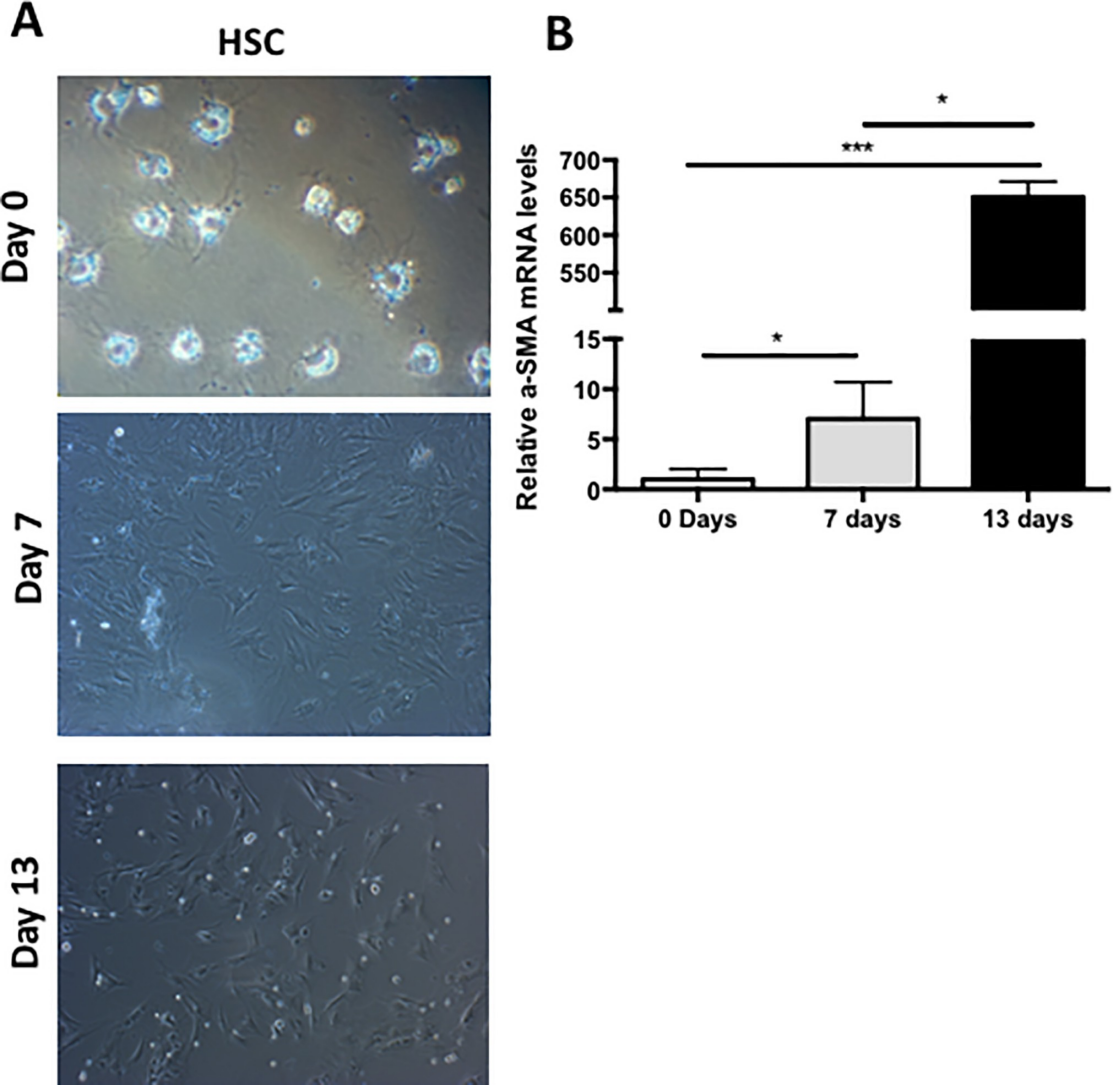
Phenotype and α-SMA expression of HSC culture. A) Representative photographs of a) freshly isolated Hepatic Stellate Cells and b) 7 and c) 13 days post-isolation. Cell auto-fluorescence, rounded shape and lipid content can be observed at day 0. In contrast, a myofibroblast-like shape was observed when cells were kept in long-term culture. B) *α-SMA* mRNA expression in Hepatic Stellate Cells at day 0, 7 and 13 post isolation. Data normalization was performed using *GAPDH* as housekeeping gene. *p<0.05.

Freshly isolated hepatic stellate cells (day zero) are slightly rounded with prominent cytoplasm and filled with lipid vacuoles (retinoids). At 7- and 13-days post-isolation cells switch to a myofibroblast-like shape phenotype and lost lipid vacuoles. Fig 1A. The expression of *α-SMA* was analyzed by RT-qPCR at 0, 7 and 13 days post-cultured, since this molecular marker is increased in activated cells. Results showed that at 7- and 13-days cells were overexpressing significantly *α-SMA* mRNA (Fig 1B; p<0.05) indicating HSC activation that correlates with phenotype changes observed in cultured (Fig 1A).

### Transfection of shRNA-CB1 inhibits CB1 expression in HSCs

**Fig 2:**
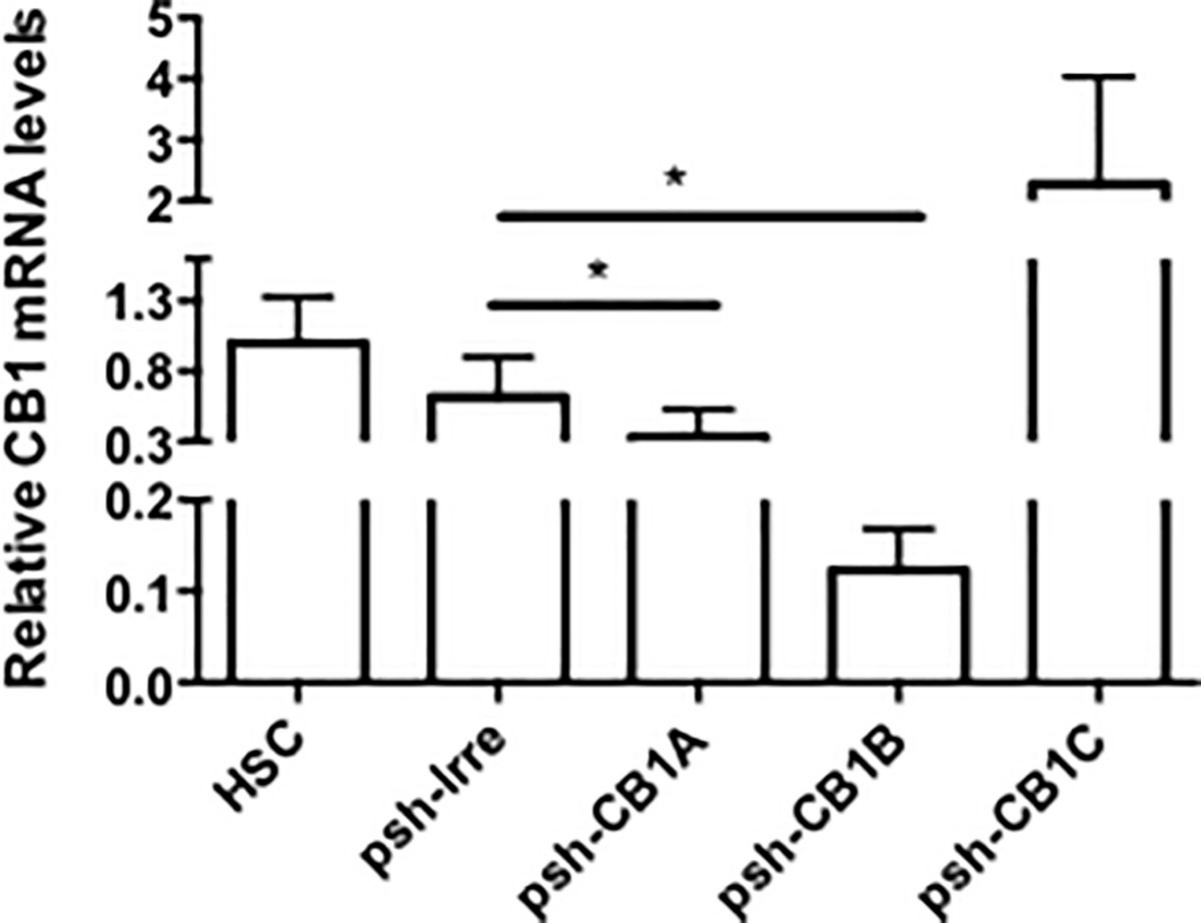
Silencing effect of shRNA-CB1 in HSCs. mRNA expression of *CB1* in hepatic stellate cells transfected with plasmid that express three different sequences for a shRNA-CB1. pshCB1-A and pshCB1-B showed a significant percentage of CB1 mRNA inhibition compared to Irrelevant shRNA. *p<0.05.

**Fig 3:**
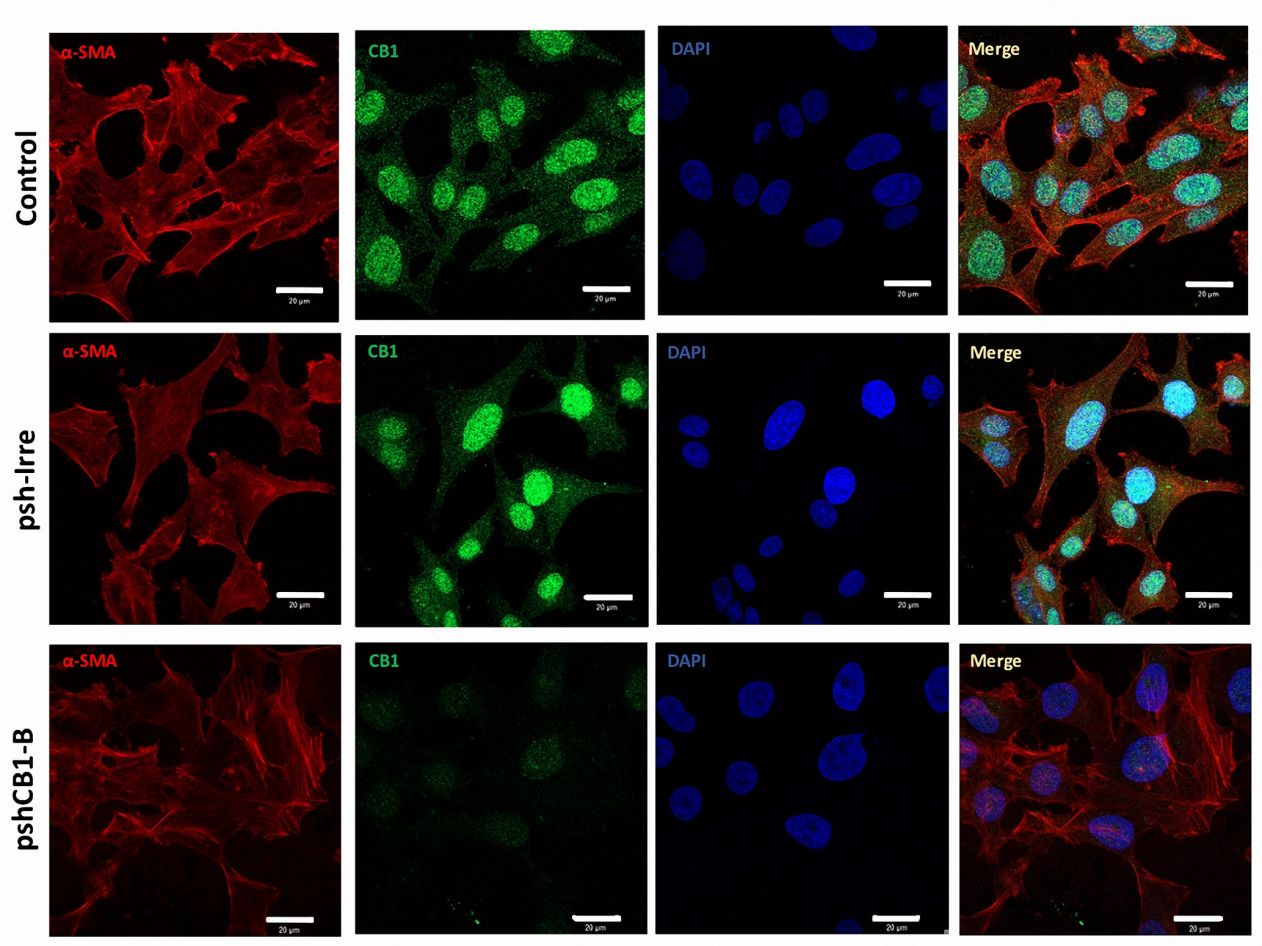
Decreased CB1 expression in cultured HSCs transfected with pshCB1-B. Representative confocal images of cultured HSCs transfected with p-Irre or p-shCB1-B. pshCB1-B demonstrated CB1 silencing compared to psh-Irre and control cells. Green: anti-CB1. Red: anti-αSMA as HSC marker. DAPI: Nuclei staining. Scale bar 20 μM.

Silencing efficacy of the designed shRNAs for *CB1* (pshCB1-A, pshCB1-B, pshCB1-C) was measured in HSC primary cultures 7 days post-isolation. *CB1* mRNA levels showed that transfection of pshCB1-A and pshCB1-B (hybridizing at position 877 and 1232) led to a significant inhibition of 62% and 85% (p<0.05), respectively; 48 hours post-transfection. Control shRNA (pshRNA-Irre) does not affect CB1 expression (Fig 2). Immunofluorescence was used to corroborate cellular inhibition of CB1 by pshCB1-B. In Fig 3 representative confocal images showed diminution in cultured HSCs transfected for 48h with pshCB1-B compared to those transfected with psh-Irre.

### pshCB1-B is the most effective shRNA blocking CB1 in the liver of cirrhotic rats

**Fig 4:**
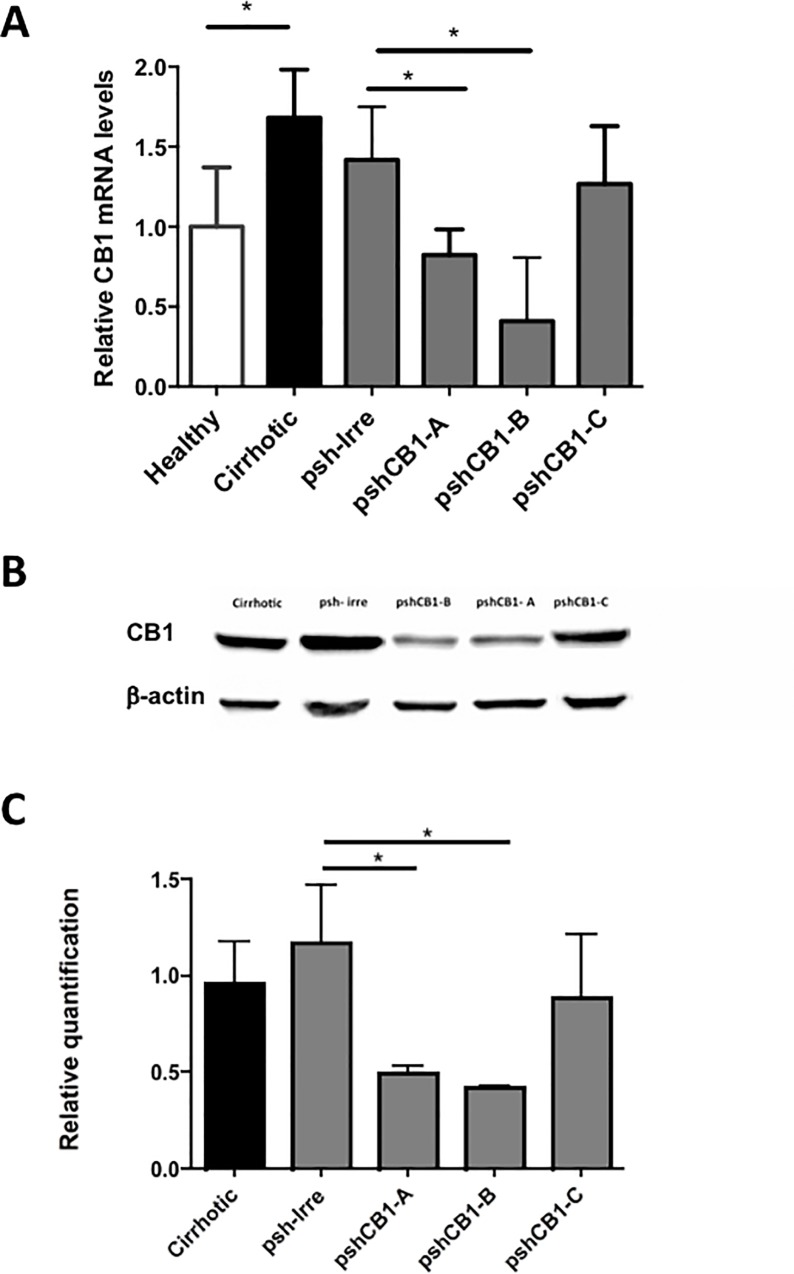
Silencing effects on CB1 expression caused by hydrodynamic transfection of shRNA-CB1 in cirrhotic animals. A) *CB1* mRNA levels showed that pshCB1-A and pshCB1-B demonstrated the major gene inhibition (*p<0.5) compared to psh-Irre control. *GAPDH* was used as housekeeping gene. B) Representative blots for CB1 protein from liver homogenates of animals transfected with shRNA-CB1. C) Densitometric analysis of three independent western blot assays for CB1 protein. Glyceraldehyde 3-phosphate dehydrogenase (GAPDH) was employed as loading control. Data are presented as the mean standard deviation. *p<0.05.

Once we confirmed that pshCB1-A and B could suppress *CB1* expression in HSCs; pshCB1-A, pshCB1-B, pshCB1-C and psh-Irre were transfected with hydrodynamic injection in the iliac vein of cirrhotic animals to validate silencing. RT-PCR of liver tissue showed that pshCB1-A and pshCB1-B markedly decreased the expression of *CB1* in treated rats, revealing that CB1 shRNA could also silence *CB1* gene *in vivo*. Also, transfection with pshCB1-B decreased *CB1* mRNA expression (55%) more than pshCB1-A that diminished it 42% (Fig 4A). Besides, CB1 western blot analysis at 96 hours post-transfection also revealed a reduce expression of protein in hepatic tissue, as depicted in Fig 4B. In a similar way, pshCB1-A and-pshCB1B significantly decreased CB1 protein expression compared with psh-Irre. A 58% and 64% diminution of the amount of this protein was observed respectively; while pshCB1-C only inhibits a 24% showing no statistical significance. Additionally, psh-Irre transfection did not showed silencing effect *in vivo*. Fig 4C. Based on these results, pshCB1-B was selected for further analysis of the effect of liver CB1 silencing in molecules involved in fibrogenesis, percentage of fibrotic tissue and liver enzymes.

### Hydrodynamic injection of pshCB1-B in cirrhotic animals did not affect CB1 expression in brain

**Fig 5:**
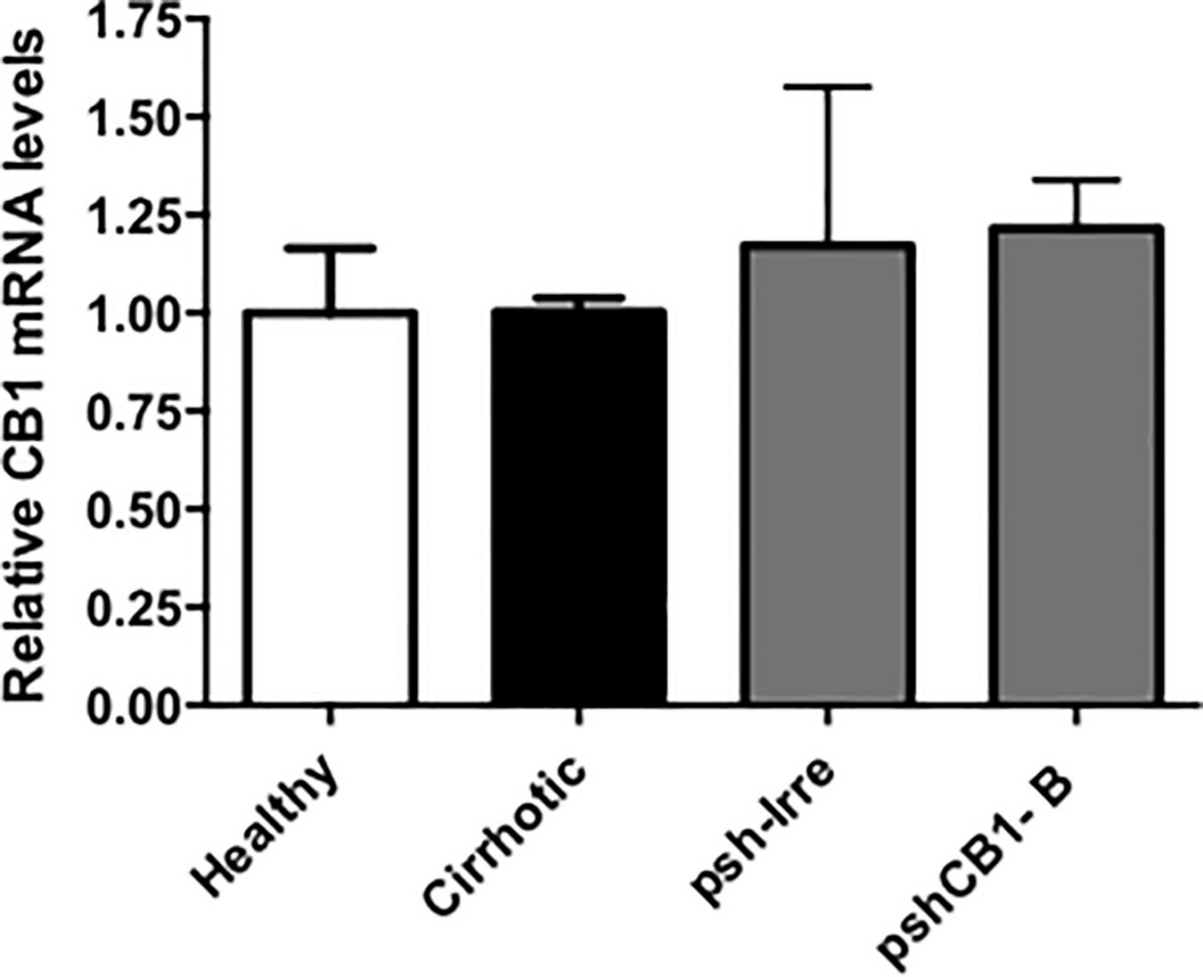
CB1 mRNA expression in brain of animals transfected with pshCB1-B. No significant differences in mRNA *CB1* levels were presented between groups. GAPDH was used to normalize the gene expression data.

As mentioned, *CB1* expression is widely distributed in SNC, and then any therapeutic approach involving its downregulation should consider not disturbing its brain expression. To test this, brain of cirrhotic animals transfected with pshCB1-B were collected. *CB1* gene expression in brain homogenate was measured by RT-PCR; results showed that mRNA expression of *CB1* remain unaffected after pshCB1-B treatment (Fig 5).

### Treatment with pshRNACB1-B reduces fibrosis and decreases TGFβ-1, Col I and α-SMA mRNA levels

**Fig 6:**
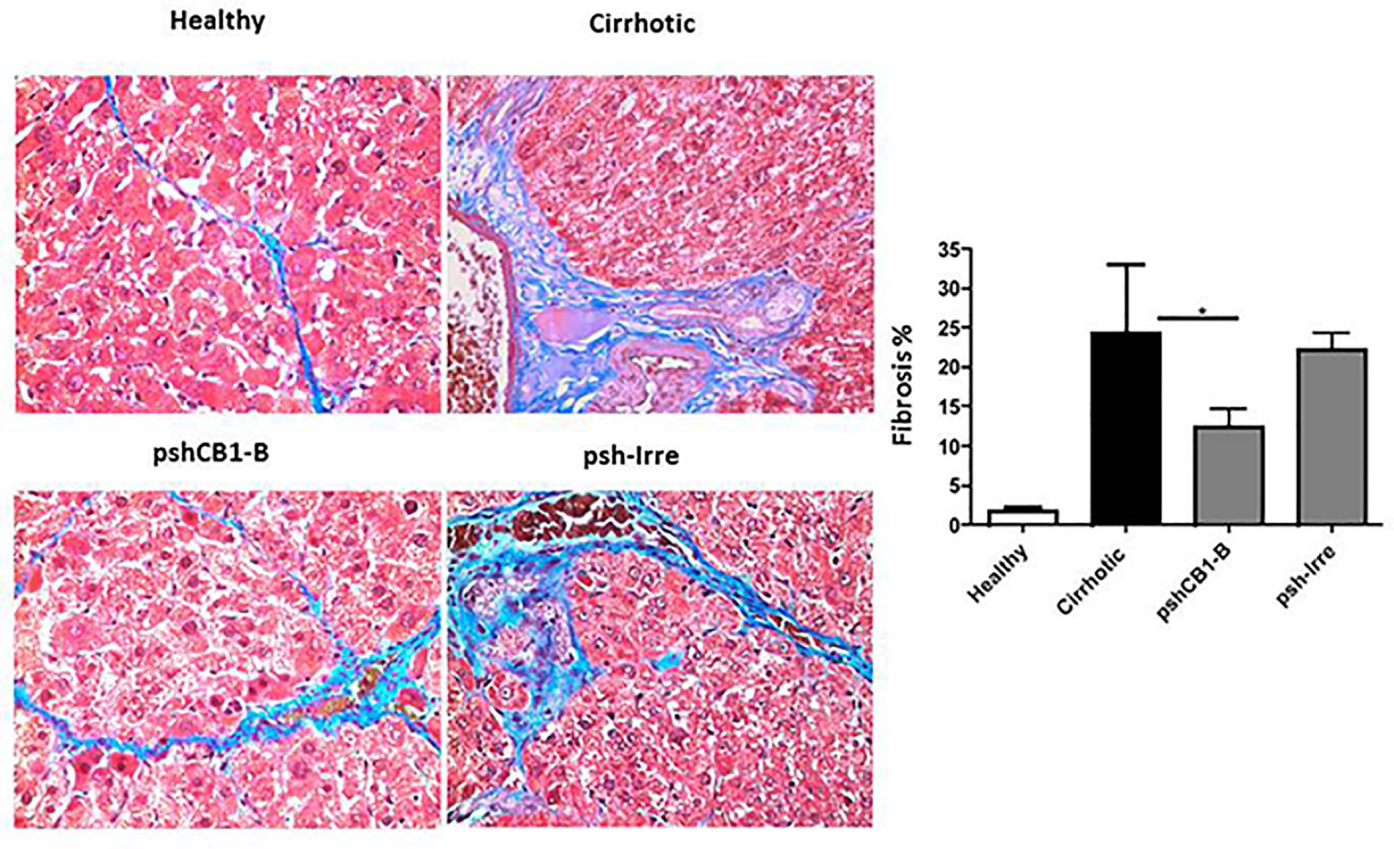
Effect of treatment with pshCB1-B on hepatic fibrosis. A) Representative microscopic photographs of liver tissue stained with Masson´s Trichrome. pshCB1-B transfection in liver markedly reduces EMC fibers stained in blue. B) Fibrosis percentage was calculated in 20 microscopic fields and data is present as mean ± the standard deviation. pshCB1-B transfected group showed a significant reduction (*p<0.05) in percentage of fibrotic tissue.

To determine the effect of pshCB1-B on hepatic fibrosis, morphometric analysis of liver sections stained with Masson´s Trichrome was performed. Non-cirrhotic control group showed normal liver histology, with scarce ECM and hepatocytes radiating outward from a central vein. Cirrhotic control and psh-Irre transfected animals showed parenchyma modifications and ECM remodeling. In contrast, pshCB1-B group showed reduced fibrosis as observed in Fig 6. Quantification analysis of stained ECM demonstrates a 24.3% and 22.3% of fibrotic tissue in cirrhotic animals administrated with vehicle and psh-Irre, respectively. pshCB1-B treated group display only 12.5% of fibrosis (p<0.05). Treatment with pshCB1-B was effective in reducing fibrotic tissue, since ECM decreased a 44% (p<0.05) comparing to irrelevant shRNA control group. Fig 6.

**Fig 7:**
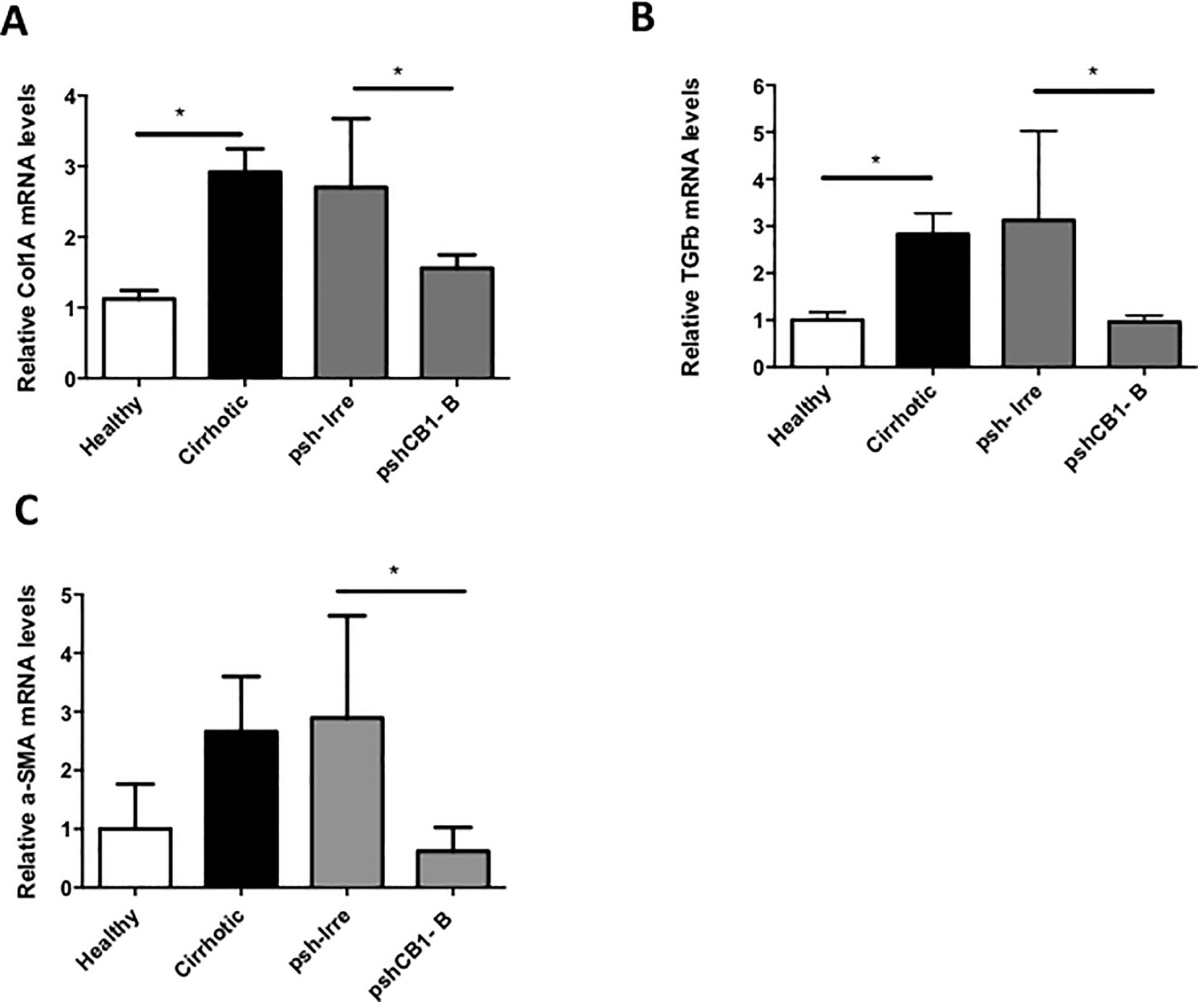
Effect of silencing with pshCB1-B on the expression of fibrogenic molecules in cirrhotic rats. A) mRNA expression of *Col IA1*, B) *TGFβ-1* and C) *α-SMA* analyzed by RT-PCR. Expression was monitored 96 hours following administration of pshCB1-B by hydrodynamic injection in the iliac vein. Expression levels of mRNA were normalized against *GAPDH*. *p<0.05.

This data correlate with Col I expression, the main ECM component increased during fibrosis. Expression of type I collagen is regulated at the transcriptional level by TGF-β1, the principal fibrogenic cytokine. Usually, high levels of TGFβ-1 on fibrotic tissue are related to high expression of Col I. Therefore, we measured mRNA expression of both in liver after transfection with pshCB1-B. As shown in Fig 7A) and 7B), pshCB1-B inhibited significantly (p<0.05) *Col IA1* mRNA (43%) and *TGFβ-1* (69%) compared to control. Finally, when HSC acquire contractibility capacity, they overexpress *α-SMA* that is used as an activation marker. mRNA analysis of *α-SMA* showed an increase of 62.43% in the amount of this mRNA in control cirrhotic group compared with healthy animals. Moreover, pshCB1-B transfection significantly decreases a 69% the expression of *α-SMA* in cirrhotic rats (Fig 7C). This could be an indirect measured of a diminution in the quantity of activated HSC.

## Discussion

Actually, there is not an established therapy for hepatic fibrosis; however, in the gene therapy area, many strategies seem promising but improvement of vectors and gene-delivery methods with higher efficiency, major selectivity for the desired tissue and minimal toxicity need to be achieved. Hydrodynamic-based transfection has been described for several authors as a safe and simple strategy for *in vivo* studies [22–24]. Hydrodynamics has showed minimal toxicity, high delivery efficiency and transient transfection to liver. Nevertheless, in recent years the approach for hydrodynamic administration has focused on reducing the injection volume but maintaining the required vascular pressure for gene transfer. In this context, injection into the vasculature of target tissues or large vessels has been successfully used [22, 25, 26]. Also, hydrodynamic had been employed for *in vivo* intracellular delivery of pDNA, siRNA, shRNA and proteins [22]. Moreover, the first time that a gene was blocked with siRNAs *in vivo* hydrodynamics was the delivery method used [3]. Based on this, we decided to test and antifibrogenic therapy using hydrodynamic administration to cirrhotic livers. Our aim is to limit transfection to hepatic tissue and silence the *CB1* gene without adverse effects in SCN. Other strategies focus on reducing the expression of *CB1* or blocking its signal transduction pathway to reduce hepatic fibrosis has been probed [27, 28]. Nonetheless, the drugs had demonstrated important behavior adverse effects and silencing using lentivirus as vectors is still not completely safe due to its random integration in the genome. Therefore, in this study we designed three shRNA to block CB1 expression and cloned them in a plasmid. To validate silencing, primary culture of HSC was transfected with the three plasmids, however only two shRNAs showed significant downregulation of *CB1*. The greater silencing effect was achieved with pshCB1-B, since *CB1* had only one exon, we believe this may be due to secondary structures formed in the mRNA that limits accessibility of the other siRNAs molecules as described by other authors [29]. To validate shRNA-mediated *CB1* gene silencing in cirrhotic animals, we used hydrodynamic transfection method to deliver shRNAs with high selectivity to the liver. In this work iliac vein was used for administration allowing direct delivery of shRNAs to liver parenchyma. Also, using this vein a reduction in volume can be done, 4ml vehicle was enough to allow efficient silencing (55%) with 750ng of plasmid. This reduction in volume improves post-administration survival compared to bigger volumes in the same vessel (data not shown), maintaining adequate vascular pressure for liver transfection. Notably, even hepatic tissue was cirrhotic (as corroborated in control cirrhotic group) transfection was efficient and as expected, shRNAs showed a similar silencing effect in hepatic tissue than that observed in HSC culture, and pshRNA-CB1-B was the most potent at mRNA and protein level.

As antifibrogenic effect, treatment with this pshCB1-B reduces notably the percentage of fibrotic tissue (almost the half of that present in cirrhotic controls) and expression of profibrogenic molecules also diminish markedly. Then, there is a correlation between Collagen expressions that decrease and fibrosis reduction in treated animals. Other authors, had also demonstrated that blocking of *CB1* mRNA reduces collagen I expression in a cirrhosis experimental model [27]. Among other crucial cytokines involved in fibrogenesis, TGF-β1 stimulates the production of Col I. pshCB1-B-mediated blocking decreased significantly *TGFβ-1* levels (P <0.05), data that are consistent with previous studies by Yang YY et al, in which treatment with AM-251 (CB1 antagonist) inhibits the hepatic expression of *TGFβ -1* in cirrhotic livers induced by bile duct ligation [13]. Also, pshCB1-B group showed a significant reduction in α-SMA mRNA. This can be translated as a possible reduction in the number of activated HSC. It has been reported that blocking CB1 (Rimonobant) induces HSC apoptosis and decreases cell proliferation; through diminution in ERK and Akt phosphorylation [14, 30].

Further analysis using this pshCB1-B demonstrated no-blocking effect in brain CB1 expression. As CB1 receptor is widely distributed in the central nervous system regulating important psycho-behavioral functions [31] other approaches for CB1 antagonism had demonstrated the inconvenience of severe behavioral side effects. Then, this shRNA sequence administrated using hydrodynamics seem to avoid these SCN secondary effects while inhibits liver expression. These results agree with Maruyama et al, who administered a plasmid by hydrodynamic injection into the tail vein, not detecting transgene expression in rat brain [6].
